# Propylthiouracil Induced Rat Model Reflects Heterogeneity Observed in Clinically Non-Obese Subjects with Nonalcoholic Fatty Liver Disease

**DOI:** 10.3390/ijms251910764

**Published:** 2024-10-07

**Authors:** Yu Jin, Qiuyan Liu, Yuqin Wang, Bing Wang, Jing An, Qimeng Chen, Tao Wang, Jing Shang

**Affiliations:** 1 School of Traditional Chinese Pharmacy, China Pharmaceutical University, Nanjing 210009, China; 2 School of Pharmacy, University of Wisconsin Madison, Madison, WI 53705, USA; 3 State Key Laboratory of Nat Mural Medicines, China Pharmaceutical University, Nanjing 210009, China; 4 Jiangsu Key Laboratory of TCM Evaluation and Translational Research, China Pharmaceutical University, Nanjing 210009, China

**Keywords:** non-obese NAFLD, propylthiouracil, lipid metabolism, animal model, carnitine metabolism

## Abstract

The prevalence of nonalcoholic fatty liver disease (NAFLD) is increasing, affecting up to 30% of the population, with approximately 20% of cases occurring in non-obese individuals. The recent shift to the term metabolic dysfunction-associated steatosis liver disease (MASLD) highlights the disease’s heterogeneity. However, there are no well-established animal models replicating non-obese NAFLD (NO-NAFLD). This study aimed to evaluate the relevance of the high-fat diet (HFD) combined with the propylthiouracil (PTU)-induced rat model in mimicking the histopathology and pathophysiology of NO-NAFLD. We first analyzed metabolic and clinical parameters between NO-NAFLD patients (Average BMI = 21.96 kg/m^2^) and obese NAFLD patients (Average BMI = 29.7 kg/m^2^). NO-NAFLD patients exhibited significantly higher levels of carnitines, phospholipids, and triglycerides. In the animal model, we examined serum lipid profiles, liver inflammation, histology, and transcriptomics. Hepatic steatosis in the HFD+PTU model at week 4 was comparable to that of the HFD model at week 8. The HFD+PTU model showed higher levels of carnitines, phospholipids, and triglycerides, supporting its relevance for NO-NAFLD. Additionally, the downregulation of lipid synthesis-related genes indicated differences in lipid accumulation between the two models. Overall, the HFD+PTU-induced rat model is a promising tool for studying the molecular mechanisms of NO-NAFLD.

## 1. Introduction

Nonalcoholic fatty liver disease (NAFLD), now being replaced by the term metabolic dysfunction-associated steatotic liver disease or MASLD [1], is the most prevalent chronic liver disease and a leading cause of liver-related morbidity and mortality. Its prevalence is estimated to be up to 30% and is continuously increasing [2,3]. Although MASLD/NAFLD is primarily considered a metabolic disease closely associated with excessive weight gain [4], it is increasingly identified in non-obese and even lean populations [5]. According to the diagnostic criteria for MASLD, individuals with a healthy body weight can still develop the disease [1]. A systematic review and meta-analysis reported that NAFLD affects up to 13.11% of lean individuals globally [6]. Within the NAFLD population, the prevalence of lean NAFLD (defined as a body mass index lower than 25 kg/m^2^) and non-obese NAFLD (NO-NAFLD) (which includes both lean and overweight individuals) is 40.8% and 19.2%, respectively [7]. Both lean and obese patients with NAFLD/MASLD share an altered metabolic and cardiovascular profile [8], but recent studies have revealed an increased mortality risk associated with lean NAFLD [9,10] and a higher incidence of gastrointestinal cancer in lean individuals with NAFLD [11].

NAFLD/MASLD represents a continuous spectrum of metabolic disease characterized by excessive lipid accumulation in hepatocytes, caused by the disruption of hepatic lipid homeostasis, defined as excessive import or diminished export and oxidation of free fatty acids. Given the complexity and heterogeneity of the disease pathogenesis [12], the lack of effective pharmacotherapeutic treatments highlights the fact that the molecular mechanisms of NAFLD, particularly NO-NAFLD, are not fully understood. Currently, animal models focus on replicating both the histopathology and pathophysiology of each stage of human NAFLD. Some models can replicate the histopathology of NAFLD without physiological properties, such as the methionine and choline (MCD) deficient diet-induced model, while others can replicate physiological properties but only mild histopathological changes, such as the high-fat diet (HFD)-induced model [13,14,15]. However, no models have accurately replicated both the histopathology and pathogenesis of NAFLD with a non-obese metabolic abnormality, which is essential for understanding NO-NAFLD pathogenesis and progression.

Therefore, we conducted this study to evaluate the relevance of a propylthiouracil (PTU) plus HFD-induced rat model, characterized by weight loss [16], for reflecting the histopathological features and pathophysiological distinction seen in clinical NAFLD or NO-NAFLD subjects. In addition, molecular mechanisms of the PTU+HFD-induced rat model were investigated by conducting a series of analyses, including liver histology, inflammation levels measured by CD68 immunofluorescence, gene expression changes in the liver measured by transcriptomics, and metabolomic changes in the liver and serum. This approach aims to facilitate the recognition of potential therapeutic responses in NO-NAFLD subtypes.

## 2. Results

### 2.1. Clinical and Biochemical Characteristics of NO-NAFLD Patients

As shown in Table 1, a total of 13 individuals were included in the study to explore distinctions between non-obese individuals with NAFLD, obese individuals with NAFLD (OB-NAFLD), and a healthy control group (HC). Non-obesity was defined as a BMI of less than 23.0 kg/m^2^ [7] Since the study aimed to capture potential distinctions between clinical groups, obesity was defined as a BMI of 28 kg/m^2^ or greater [7], while non-obese individuals were defined as having a BMI less than 23.0 kg/m^2^ [17]. Subjects with a BMI between 23 kg/m^2^ and 28 kg/m^2^ were not included in the study. The average BMI for the NO-NAFLD group (*n* = 3) was 21.96 kg/m^2^, compared to 29.70 kg/m^2^ for the OB-NAFLD group (*n* = 5), and 23.50 kg/m^2^ for the HC group (*n* = 5). No significant differences were observed regarding basic demographic, clinical, and laboratory characteristics (Table 1) between the NAFLD groups. 

### 2.2. Distinctive Serum Metabolites Characteristics Were Identified between Clinical NO-NAFLD and OB-NAFLD Subjects

**Figure 1:**
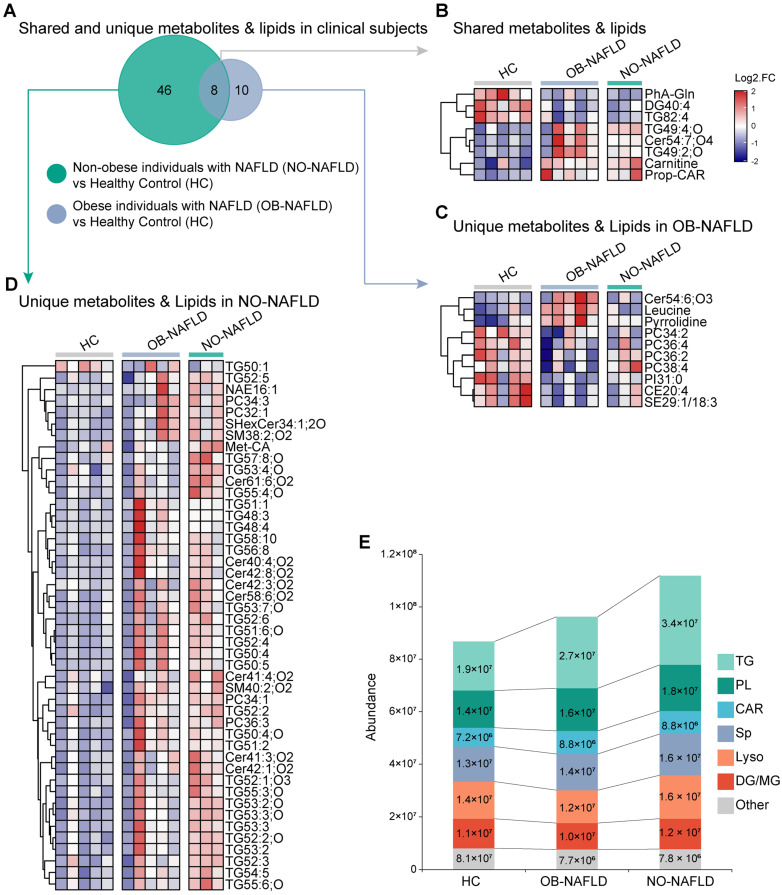
Metabolomic and lipidomic profiling of serum metabolites reveal distinct signatures between NO-NAFLD and OB-NAFLD patients. (**A**) illustrates the distribution of shared and unique serum metabolites in OB-NAFLD and NO-NAFLD patient groups compared to HC; (**B**) highlights the shared metabolites and lipids between OB-NALFD and NO-NAFLD compared to HC; (**C**,**D**) displays the unique metabolites and lipids observed in OB-NAFLD groups compared to HC and NO-NAFLD compared to HC, respectively; (**E**) depicts the total abundance distribution changes of various fatty acid carrier substances. Abbreviations: TG, triglycerides; PL, phospholipids; CAR, carnitines; Sp, sphingolipids; Lyso, lysophospholipids; DG/MG, diacylglycerols/monoglycerides.

The results from metabolomics and lipidomic analyses implied that distinctions in metabolomics between NO-NAFLD and OB-NAFLD existed (Figure 1A), which were confirmed by sub-analysis (Figure 1A). Both groups displayed prominently elevated serum carnitine metabolites and reduced levels of serum phenylacetylglutamine compared to the HC group (Figure 1B). A decreased concentration of leucine was observed in NO-NAFLD, contrasting with a significant increase in OB-NAFLD (Figure 1C). Importantly, the NO-NAFLD group had significantly higher levels of certain serum metabolites compared to the OB-NAFLD and HC groups, including serum triglycerides (TG52:5, TG57:8;O, TG53:4;O) and phospholipids (PC 36:3, PC 34:3, PC 34:1, PC 32:1) (Figure 1D).

Given the importance of carnitines, phospholipids, and triglycerides in fatty acid transportation, we examined other lipid classes associated with this process. Significantly higher concentrations of carnitines, phospholipids, and triglycerides in NO-NAFLD subjects were reinforced (Figure 1E).

### 2.3. The Pathological Features Characterized in the HFD+PTU-Induced NAFLD Rat Model

**Figure 2:**
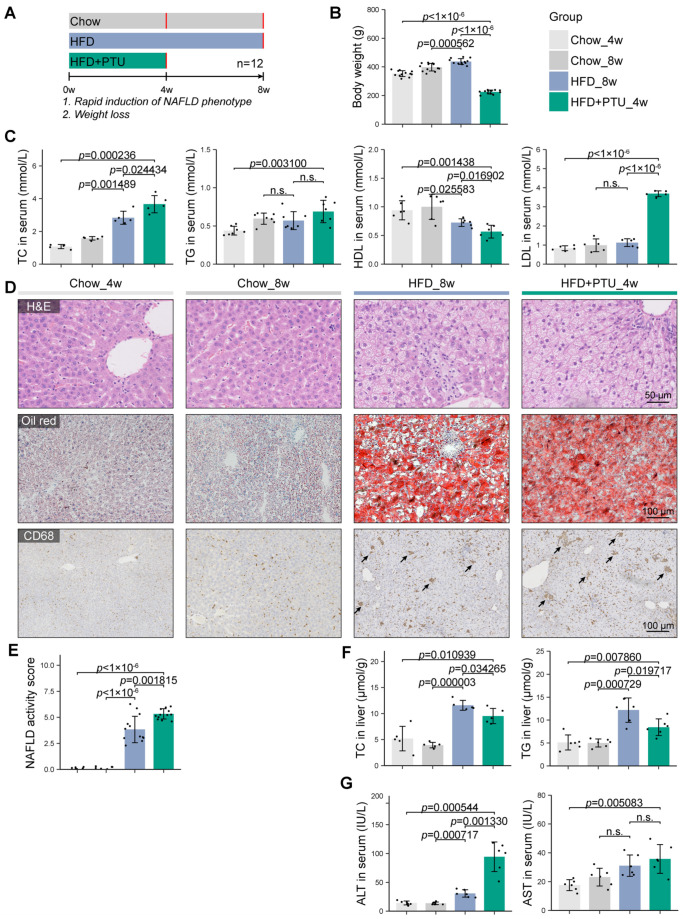
Cross-comparisons of phenotypical characteristics among the HFD+PTU-induced rat model, the HFD-induced rat model, and the chow group. (**A**) Schematic diagram illustrating the process of establishing the HFD+PTU-induced model and the HFD-induced model, with *n* = 12 per group (**B**) Body weights of the subjects; (**C**) Lipid panel results; (**D**) Histological features of hepatic tissue sections stained with H&E, Oil Red O, and CD68 immunohistochemistry. The arrows point to the inflammation foci. (**E**) NAFLD activity score; (**F**) Concentration of TC and TG in the liver; (**G**) Concentration of serum ALT and AST were obtained and analyzed from no fewer than 6 rats per group. Abbreviations: NAS, NAFLD activity score; n.s., non-significant.

We compared the HFD+PTU-induced rat model at week 4 (HFD+PTU_4w) to both the HFD-induced rat model at week 8 (HFD_8w) and the chow diet group at both week 4 and 8 to assess its relevance in replicating the phenotypical characteristics of clinical NAFLD (Figure 2A). A statistically significant reduction in body weight was observed in HFD+PTU_4w (Figure 2B) and the MCD-induced mouse group at week 4 (Figure S2) used as a weight loss model comparison. The lipid profile of HFD+PTU_4w demonstrated a more severe metabolic condition stimulated with significantly higher concentrations of serum TC and TG (Figure 2C) compared to the HFD_8w. Unlike HFD+PTU_4w, the MCD-induced group showed decreased concentrations of serum TG, TC, and LDL (Figure S2), affirming its limitation in presenting the metabolic profile of clinical NAFLD.

To investigate hepatic steatosis and inflammation in the HFD+PTU-induced rats, liver sections stained with Hematoxylin and Eosin (H&E), Oil Red, and CD68 immunohistochemistry were examined and evaluated among the groups. Steatosis with obvious lipid accumulation and an increased number of CD68+ cells were evident in HFD+PTU_4w and HFD_8w (Figure 2D). A significantly higher NAFLD activity score (NAS) indicated a severe stage of NAFLD induced by the HFD+PTU method (Figure 2E). Additionally, significantly increased concentrations of TC and TG in the liver, along with serum ALT and AST, were observed in the HFD+PTU_4w compared to the chow group (Figure 2F,G).

### 2.4. The Consistency of Metabolites Characteristics between HFD+PTU-Induced Rat Model and Clinical NO-NAFLD Subjects

**Figure 3:**
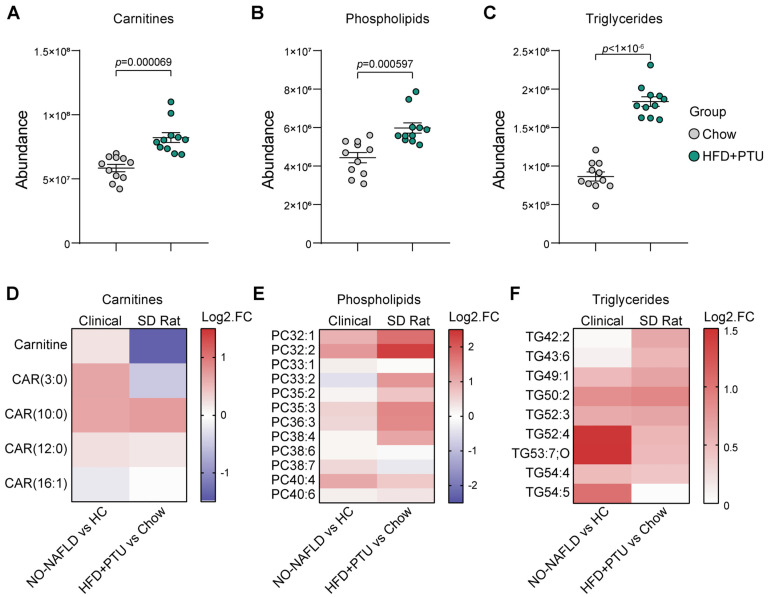
Similarity analysis of the concentration changes of carnitines, phospholipids, and triglyceride metabolites between the HFD+PTU-induced model and NO-NAFLD clinical subjects. (**A**–**C**) show the alterations in the total amounts of carnitine, phospholipid, and triglyceride metabolites in the HFD+PTU-induced rat model, with *n* = 11 per group; (**D**–**F**) provide a parallel comparison of the specific compositional changes in carnitines, phospholipids, and triglycerides between the NO-NAFLD patients vs. HC, and the HFD+PTU-induced model vs. chow. Red indicates upregulation, and blue indicates downregulation.

We conducted metabolomics analysis on HFD+PTU-induced rats and a control group. A statistically significant higher abundance of total carnitines, phospholipids, and triglycerides was observed in the HFD+PTU group (Figure 3A–C). However, the concentration of carnitine and short-chain acylcarnitine (CAR 3:0) was lower compared to the chow group (Figure 3D). This discrepancy might be characteristic of rodent models, as a lower concentration of carnitine was also observed in the HFD-induced rat model (Figure 1C,D). Various types of phospholipids and triglycerides were further analyzed, with results shown in Figure 3E,F, endorsing the consistency. These findings suggest the similarity in pathogenic mechanisms shared between the HFD+PTU rat model and NO-NAFLD subjects.

### 2.5. Liver Transcriptomes Analysis of the HFD+PTU-Induced Rat Model

**Figure 4:**
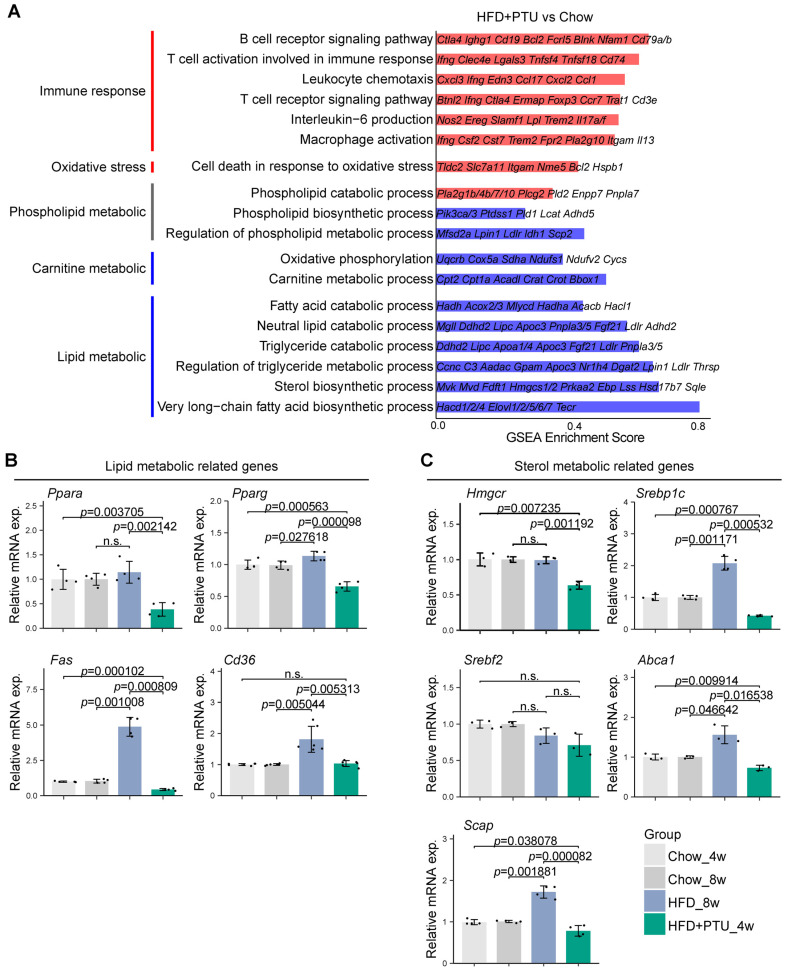
Transcriptome analysis reveals that compared with the traditional high-fat diet (HFD)-induced model, the HFD+PTU-induced rat model exhibited unique metabolic functional changes in the liver, with distinct characteristic mechanisms, with *n* = 4 per group. (**A**) GSEA illustrating changes in the expression of pathways and genes related to immune activation, phospholipid and carnitine metabolism, and lipid metabolism in the liver of the HFD+PTU model, with red indicating upregulation and blue indicating downregulation; (**B**) RT-qPCR results showing differences in the expression of genes related to lipid metabolism, with *n* ≥ 3 per group, (**C**) RT-qPCR results showing differences in the expression of genes related to sterol metabolism, with *n* ≥ 3 per group. Abbreviation: GSEA, Gene Set Enrichment Analysis; n.s., non-significant.

A comprehensive analysis of the hepatic transcriptome was conducted to explore the mechanisms underlying HFD+PTU-induced NAFLD, focusing on the causes of lipid accumulation in the liver. Genes and pathways related to immune response and oxidative stress (*Bcl2*, *Cxcl3*, *Trem2*) concerning NAFLD were examined using Gene Set Enrichment Analysis (GSEA). Significant upregulation of these genes, along with increased mRNA expression of *CD68*, supported the relevance of the HFD+PTU-induced rat model. Pathways associated with phospholipid synthesis were significantly downregulated, while those related to phospholipid degradation were significantly upregulated. Additionally, the expression of genes related to carnitine metabolism showed a significant downtrend (Figure 4A).

From the GSEA, multiple pathways associated with lipid and sterol catabolic processes were downregulated, including those related to fatty acid catabolism, triglyceride catabolism, and the regulation of triglyceride metabolism (Figure 4A). Next, we examined the mRNA expression of genes strongly associated with lipid and sterol metabolic processes as well as the progression of hepatic steatosis. Notably, the expression of all selected genes was significantly diminished at various levels compared to the control group, while most genes showed a significant increase in the HFD group at week 8, such as *Fas Cd36*, *Srepb1c*, and *Abca1* (Figure 4B,C). Taken together, the inhibition of lipid and sterol degradation and the subsequent reduction in energy expenditure observed in the HFD+PTU-induced model led to fat accumulation in the liver. This contrasts with the HFD-induced model, where stimulation of lipid synthesis is the predominant mechanism.

## 3. Discussion

With the increasing prevalence of NAFLD in non-obese populations globally [7], an accurate model to characterize the lean metabolic features of this subgroup remains undefined. This study provides evidence that the HFD+PTU-induced rat model effectively characterizes the lean metabolic features of NO-NAFLD subjects. Compared to the HFD-induced rat model, it took a shorter duration to develop histologically proven steatosis, making the HFD+PTU model more applicable, and the model exhibits distinctive mechanisms of hepatic steatosis driven by the inhibition of lipid disposal. The integration of metabolomics, lipidomic, and transcriptomic analyses enhances the reliability of the results.

Histopathology is the gold standard for clinical diagnosis and is equally important in experimental settings [13,18]. The NAS was developed to facilitate the diagnosis of NAFLD or NASH in humans and to semi-quantify the stage of disease [19]. As shown in Figure 3, the HFD+PTU-induced model effectively developed representative histological features of NAFLD. Meanwhile, mild steatosis in HFD_8W, along with significantly higher NAS scores in HFD+PTU compared to HFD_8W, indicates that the HFD+PTU model accelerated the development and progression of the disease. Metabolic syndrome is a crucial pathogenic determinant that an optimal experimental model should reflect [20]; dyslipidemia with weight loss effects observed in the HFD+PTU-induced model demonstrates its relevance in replicating NO-NAFLD subjects. Moreover, the HFD+PTU-induced rat model, when compared to the MCD-induced model featuring weight loss [13], confirmed its uniqueness in capturing the lean metabolic features of NO-NAFLD subjects. The MCD model failed to produce a sufficient metabolic response and was supported by other studies because the deficiency of choline in diets, which impacts hepatic very-low-density lipoprotein secretion and lipid peroxidation in hepatocytes [21], and lack of metabolic alternation as pathogenic mechanisms are localized to the liver [22,23]. The HFD+PTU-induced model successfully replicates the key histopathological features of NAFLD and lean metabolic abnormalities captured from NO-NALFD subjects.

Pathophysiological and genetic changes play an essential role in experimental models [20]; metabolomics will help elucidate disease progression mechanisms by identifying the link between genotype and phenotype [24]. Few studies have reported the relationship between increased metabolites and NAFLD sub-types, but significantly increased plasma concentrations of total carnitines in subjects with steatosis or NASH have been reported [25,26]. The significantly increased total carnitine metabolites reported in the NO-NAFLD subjects, according to other studies, may reflect altered mitochondrial function, reduced carnitine turnover, and decreased fatty acid oxidation [27,28]. Carnitines play an essential role in transporting fatty acids into mitochondria; the concentration of fatty acylcarnitine in the liver reflects the extent of fatty acid oxidation [27,29]. As a result, a reduced fatty acid utilization rate could lead to a compensatory increase in triglycerides and phospholipids [28,30], consistent with the study observation as well (Figure 1).

In the HFD+PTU-induced rat model, significantly increased metabolites of carnitines, phospholipids, and triglycerides were observed compared to the chow group (Figure 3). And associated genes and pathways were further tested by transcriptomic analysis. Fat accumulation in the liver was driven by the inhibition of lipid disposal rather than the activation of acquisition or de novo lipogenesis (DNL) induced by a high-fat diet. The upregulated genes and pathways associated (Figure 4) with fat accumulation in the liver of the HFD-induced model were consistent with the literature [23]. Excessive fat intake stimulated the DNL pathway by upregulating key enzymes genes such as *Fas* and *Hmgcr*, involved in fatty acid and cholesterol synthesis, as shown in Figure 4. However, in the HFD+PTU-induced model, diminished expression of *Fas* and *Hmgcr* indicated suppression of the DNL pathway, along with the downregulation of *Ppara*, suggesting an altered fat accumulation mechanism from the HFD-induced rat model.

Comparing omics data from human and animal models helped validate the pathophysiological relevance of the HFD+PTU model [31]. For clinical data analysis, the collaboration of metabolomics and lipidomic analyses helped narrow down the distinction between groups into a metabolomic profile (Figure S1). Since human samples are difficult to collect, transcriptomic analysis was only applied to experimental animals. In this study, transcriptomic analysis complemented with metabolomics analysis was an essential step to demonstrate that the increased metabolites were associated with genes and pathways linked to fat accumulation in the liver.

In addition, it is important to highlight several related observations that contribute to the understanding of the mechanisms of the HFD+PTU-induced model and the uniqueness of NO-NALFD. First, while a link between hypothyroidism and NAFLD has been suggested [32,33], few studies have explored the relationship between thyroid function and NO-NAFLD. One review found that thyroid-stimulating hormone (TSH) levels in lean NAFLD subjects were significantly higher than in OB-NAFLD [34]. Given the anti-hyperthyroidism nature of PTU, which affects TH levels, impacting lipid metabolism [35]. Therefore, altered TH might link to the lean metabolism abnormalities observed in HFD+PTU-induced rats, as studies have reported weight loss in rats induced by PTU regardless of a high-fat diet [36,37]. Secondly, non-significant altered leucine levels observed in NO-NALFD may reflect a difference in insulin sensitivity between groups [38,39]. Leucine, a branched-chain amino acid (BCAA), has been reported to have increased concentrations in clinical NAFLD [25], aligning with observations from obese-NAFLD subjects but contrasting with the NO-NAFLD group. Additionally, the non-increased leucine level supports the notion that hepatic fat accumulation in NO-NAFLD subjects is primarily driven by the suppression of fatty acid disposal pathways, as leucine, a BCAA, stimulates skeletal muscle to synthesize short-chain fatty acyl carnitine [40], which is not consistent with our observations. Lastly, as shown in Figure 3, carnitines and propionylcarnitine in the HFD+PTU-induced group were decreased instead, but this has also been reported in PTU-induced hypothyroidism models [41], HFD-induced models, and other NAFLD rodent models [42], suggesting that this inconsistency is driven by various factors rather than PTU alone

Limitations of this study include that only a few subjects with NO-NAFLD were included. Since the PTU can decrease the concentration of TH, TH should be tested in both clinical subjects and experimental animals as well as other metabolic features such as insulin sensitivity. Further research should investigate the molecular link between thyroid hormone (TH) and NO-NAFLD. Exploring the HFD+PTU model’s translatability to human conditions and its potential for drug response studies is also recommended.

## 4. Materials and Methods

### 4.1. Patients Selection 

The participants’ health records were retrieved from the Health Management Center of the Second Affiliated Hospital of Guilin Medical University between September and October 2023. A total of 201 individuals were selected, including 96 in the NAFLD group and 105 healthy controls. Detailed inclusion and exclusion criteria are displayed in Table S1. NAFLD patients were stratified into non-obese (BMI < 23.0 kg/m^2^, *n* = 3) and obese (BMI ≥ 28.0 kg/m^2^, *n* = 5, age/sex-matched) subgroups. Five healthy controls without fatty liver served as the N group.

### 4.2. Animal Processing Methods

A total of 48 Sprague Dawley rats (180–220 g, SCXK(HU)2013-0016) were randomized by body weight into HFD, HFD+PTU, and control groups with 12 rats per group. They were treated with either HFD (88% chow, 10% lard, 2% cholesterol) for 8 weeks (HFD group) or HFD+0.2% PTU (72.8% chow, 10% lard, 10% sucrose, 2% cholesterol, 5% egg yolk, 0.2% PTU) for 4 weeks (HFD+PTU group). The control group was fed by chow diet. Body weight and food intake were recorded weekly.

Six-week-old male C57BL/6 mice (SCXK(SU)2021-0013), housed in specific pathogen-free (SPF) conditions (SYXK(SU)2021-0011) at 22–24 °C with a 12 h light/dark cycle, were randomized by body weight into the MCD (TP3005G, TROPHIC) group or chow diet (LAD3001G, TROPHIC) group with 12 mice per group after a 2-week acclimatization period. A 4-week MCD regimen was employed to develop NAFLD phenotypes.

### 4.3. Biochemical Parameters Measurement

#### 4.3.1. Clinical Samples

Fasting venous blood of the enrolled clinical subjects was collected in the morning (between 08:00 am–12:00 pm) for lipid panel, glucose, and liver function assessment by using Cobas 8000 (Roche). The remaining samples were stored at −80 °C for metabolomic analysis.

#### 4.3.2. Animal Samples

Experimental animals were fasted overnight and then euthanized in the morning (between 08:00 am–12:00 pm) via cervical dislocation. Blood from the orbital venous plexus and tissues were collected promptly for analysis or stored at −80 °C.

All protocols involving animal experiments were approved by the Animal Care and Use Committee of China Pharmaceutical University and conformed to the Guide for the Care and Use of Laboratory Animals (8th edition)

### 4.4. Metabolomic Analysis

#### 4.4.1. Polar Metabolites Extraction

180 μL of pre-cooled methanol (containing 200 ng/mL *p*-chlorophenylalanine as an internal standard) was added to 30 μL of serum from clinical samples or experimental animals, then mixed and vortexed well. The metabolites were extracted by ultrasonication in an ice bath for 5 min. The mixture was then left to stand for 1 h and centrifuged at 13,300 rpm at 4 °C for 15 min. The supernatant was vacuum-dried using a SpeedVac Concentrator (Thermo Fisher Scientific, Waltham, MA, USA) and reconstituted with 100 μL of a 60% acetonitrile (ACN) aqueous solution. Before analysis, the prepared sample was stored at −20 °C

#### 4.4.2. Lipid Extraction

To 30 μL of serum from clinical samples or experimental animals, 180 μL of methanol was added, vortexed, and mixed. Then, 600 μL of methyl tert-butyl ether was added and shaken at room temperature for 1 h. Next, 150 μL of water was added, and the mixture was left to stand at room temperature for 10 min. The mixture was then centrifuged at 13,300 rpm at room temperature for 15 min. The supernatant was dried using a SpeedVac. The dry residue was dissolved in 300 μL of solvent (ACN/isopropanol/water = 60:35:5, containing 200 ng/mL of lysophosphatidylcholine (17:0) as an internal standard) with ultrasonication in an ice bath for 5 min. The reconstitution solution was then centrifuged at 13,300 rpm at room temperature for 10 min. The supernatant was used for further analysis.

#### 4.4.3. HPLC-MS

Metabolite analyses were performed using 1260 Infinity high-performance liquid chromatography (HPLC) coupled with 6530 quadrupole-time-of-flight (qTOF) mass spectrometry (MS) (Agilent Technologies, Santa Clara, CA, USA). Chromatographic separation of metabolites was conducted using an HSS T3 column (2.1 × 100 mm, 2.5 μm) at 40 °C.

For polar metabolite analysis, the mobile phases consisted of (A) water with 0.1% formic acid and 10 mM ammonium formate, and (B) ACN, both at a flow rate of 0.4 mL/min. The liquid chromatography gradient was held at 98% A for 2 min, then decreased from 98% to 40% A from 2 to 7 min, from 40% to 0% A from 7 to 20 min, and held at 0% for 3 min. The gradient then returned to the initial setting over a post time of 7 min.

For lipid analysis, the mobile phases consisted of (A) water = 4:6 (containing 0.1% formic acid and 10 mM ammonium formate), and (B) ACN = 1:9, both at a flow rate of 0.4 mL/min. The liquid chromatography gradient was held at 75% A for 2 min, then decreased from 75% to 35% A from 2 to 5 min, from 35% to 5% A from 5 to 23 min, from 5% to 1% A from 23 to 31 min, and held at 1% for 3 min. The gradient then returned to the initial setting over a post time of 6 min.

Ion acquisition was conducted in positive mode with a scanning range from 100 to 1700 m/z. The ion source parameters were set as follows: drying gas at 325 °C with a flow rate of 10 L/min; nebulizer at 40 psig; capillary voltage at 4000 V; fragmentor at 130 V; skimmer at 65 V; and octopole radio frequency (RF) peak at 750 V.

#### 4.4.4. Data Processing

The data were processed using MS-DIAL (v5.1.230912) for peak extraction, alignment, differential comparison, and annotation based on fragment ion information from parent ions. The identified polar metabolites and lipid ions were then integrated for further visualization using the ggplot2 R package (v3.5.1).

### 4.5. RNAseq Analysis

Total RNA was extracted from liver samples, and its purity, concentration, and integrity were verified using a NanoPhotometer^®^ and Agilent Bioanalyzer 2100. Enrichment of mRNA with oligo(dT) magnetic beads and fragmentation of mRNA was followed by synthesis of the first and second cDNA strands by using a NEBNext^®^ UltraTM RNA Library Prep Kit. After purification, cDNAs were repaired, adenylated, and adaptor-ligated for size selection. PCR amplification was performed to construct the sequencing library, which was conducted, quality-checked, and sequenced on an Illumina Novaseq platform.

Raw FASTQ reads were processed using custom Perl scripts to remove adapters, poly-N sequences, and low-quality reads, resulting in clean data. Quality metrics including Q20, Q30, and GC content were assessed. Clean reads were mapped to the reference genome using Hisat2 v2.0.5, leveraging gene model annotations for improved splice junction detection. Gene expression levels were quantified as FPKM values using featureCounts v1.5.0-p3. Differential expression analysis was performed with DESeq2 (v1.16.1), adjusting *p*-values using the Benjamini–Hochberg method. Genes with adjusted *p* < 0.05 were considered differentially expressed.

### 4.6. RNA Isolation and Quantitative Real-Time PCR

Total RNA was extracted using a Trizol reagent (Invitrogen, Carlsbad, CA, USA). Reverse transcribing involved a cDNA synthesis kit (Takara Bio, Kusatsu, Japan). Quantitative PCR (qPCR) involved Power SYBR green PCR master mix (Takara Bio, Kusatsu, Japan) and RT-PCR reverse transcriber-Veriti 96 (Applied Biosystems, Waltham, USA); real-time fluorescent quantitative PCR amplifier-7500 (Applied Biosystems, Waltham, USA) with GAPDH as an endogenous control. Primer sequences for qPCR were from PrimerBank.

### 4.7. Experimental Model Liver Histology

Experimental liver tissues were fixed in 4% paraformaldehyde for 24 h at room temperature, then paraffin-embedded and sectioned at 8 μm. HE staining was performed on paraffin sections for histological assessment. Fresh tissues were OTC-embedded and stained sequentially with Oil Red and hematoxylin for lipid visualization. Whole-mounted liver tissues, fixed via vascular perfusion with 4% PFA in PBS, were blocked with 5% BSA/0.3% PBS-T for 1 h at RT. Overnight incubation with CD68 antibodies (1:800, Abcam, ab201340) at 4 °C was followed by secondary antibody labeling with HRP at RT for 1 h. Signals were visualized using an Olympus SZX16 stereoscope, and images were captured for analysis.

### 4.8. Statistic Analysis

All data are presented as mean ± standard deviation (SD). Comparisons between treatment groups and controls were conducted using a one-way analysis of variance (ANOVA) followed by Tukey’s test in GraphPad PRISM, with statistical significance set at *p* < 0.05. For the heatmaps in Figure 1, they were performed using the ggplot2 R package (v3.5.1), in which the color of each cell represented the value of various indices normalized by row in individual samples. And for the heatmaps in Figure 3, they were performed using GraphPad PRISM, in which the color of each cell represented the foldchange of various indices calculated with the mean of each group.

## 5. Conclusions

Our study demonstrates the significance of the HFD+PTU-induced rat model in resembling key features of NO-NAFLD subjects, including distinctive metabolite characteristics, lipid profile, lower body weight, and typical steatosis characteristics seen in clinical NAFLD. The matching alterations in carnitines, phospholipids, and triglyceride levels with clinical observations underscore its applicability. Moreover, the relevance of the HFD+PTU-induced rat model was confirmed by both metabolomics and hepatic transcriptomic analyses. The model successfully simulated an advanced stage of NAFLD within four weeks, highlighting its potential for studying NO-NAFLD.

## Figures and Tables

**Table 1:** Basic demographic, clinical, and laboratory characteristics of the clinical subjects.

Characteristic	HC (*n* = 5)		OB-NAFLD (*n* = 5)		NO-NAFLD (*n* = 3)		*p* Value OB-NAFLD vs. HC	*p* Value NO-NAFLD vs. HC	*p* Value NO-NAFLD vs. OB-NAFLD
	Mean	SD	Mean	SD	Mean	SD			
Age (year)	43.00	9.72	38.40	5.90	51.00	7.00	0.398	0.230	0.064
Sex (Female, %)	20.00%	-	20.00%	-	33.33%	-	-	-	-
Body weight (kg)	61.80	7.08	84.26	7.12	58.73	3.40	0.001 *	0.448	0.001 *
BMI (kg/m^2^)	23.50	2.52	29.70	1.36	21.96	1.66	0.003 *	0.344	0.003 *
FBG (mmol/L)	4.90	0.51	5.12	0.70	5.25	0.55	0.601	0.432	0.784
ALT (U/L)	11.60	3.21	31.20	6.76	23.67	3.51	0.001 *	0.008 *	0.084
AST (U/L)	18.10	1.95	22.80	4.97	23.33	1.53	0.103	0.007 *	0.832
CHOL (mmol/L)	4.70	0.42	5.17	0.68	5.48	0.90	0.240	0.268	0.631
TG (mmol/L)	0.90	0.36	2.63	2.31	2.25	0.44	0.169	0.013 *	0.737
LDL(mmol/L)	3.00	0.41	3.36	0.82	3.69	0.89	0.369	0.287	0.624
HDL (mmol/L)	1.40	0.11	1.09	0.26	1.15	0.34	0.063	0.353	0.813

Table abbreviations: HC, healthy control; OB, obese; NO, non-obese; BMI, body mass index; FBG, fasting blood glucose; ALT, alanine transaminase; AST, aspartate aminotransferase; CHOL, cholesterol; TG, triglycerides; LDL, low-density lipoprotein; HDL, high-density lipoprotein. * Denotes statistical significance at *p* < 0.05.

## Data Availability

Data are contained within the article and Supplementary Materials.

## Supplementary Materials

The supporting information can be downloaded at https://www.mdpi.com/article/10.3390/ijms251910764/s1.
