# Supplementary material for: Propylthiouracil Induced Rat Model Reflects Heterogeneity Observed in Clinically Non-Obese Subjects with Nonalcoholic Fatty Liver Disease
Source: Int J Mol Sci. 2024 Oct 7;25(19):10764. doi: 10.3390/ijms251910764 (PMC11477315; doi:10.3390/ijms251910764)
Supplement: Supplementary file 1 [file ijms-25-10764-s001.zip › ijms-3181393-supplementary.pdf]

Supplemental material

Clinical sample

Diagnosis of NAFLD

The diagnostic criteria [1] for NAFLD included: (1) the presence of hepatic steatosis by imaging of abdominal ultrasonography; (2) a history of no or limited daily alcohol consumption (< 20 g for women and < 30 g for men); and (3) the exclusion of all other liver diseases and other abnormal condition to induce hepatic steatosis such as viral hepatitis, autoimmune hepatitis, drug-induced hepatitis, Wilson's disease, Cushing's syndrome and hypothyroidism.

Laboratory examination

Fasting venous blood from the subjects collected from 8:00 to 12:00 was used for biochemical analysis. Serum lipid, blood glucose and liver function related indictors were detected by automatic biochemical analyzer (Cobas 8000, Roche, Basel, Switzerland). The remained serum samples were stored at -80 ℃ until further analysis of metabolite.

Questionnaire survey

To collect general health data, a questionnaire involving history of illness, medication treatment, alcohol consumption, smoking, and status of exercise, sleep and diet was provided for participants.

Table S1 Detailed inclusion and exclusion criteria for participants

**Detailed inclusion and exclusion criteria for participants**	
**Inclusion criteria**	
**For all participants**	
1. Age of 18 to 65.	
2. Participants are aware of the research content and agree to write informed consent.	
**For health participants**	**For NAFLD patients**
1. Body weight > 50 kg for men, > 45 kg for women, and BMI between 20 and 25 kg/m^2.	1. Meet the diagnosis criteria of **Guidelines of prevention and treatment for nonalcoholic fatty liver disease: A 2018 update**.
**Exclusion criteria**	
**For all participants**	
1. Women who are pregnant or lactating.	
2. Previous confirmed mental illness or use of psychiatric medications and antidepressants.	
3. Diabetes mellitus other than type 2 diabetes mellitus.	
4. Taking hypoglycemic, lipid-lowering agents currently.	
5. A history of liver cirrhosis, hepatic encephalopathy or liver transplantation.	
6. History of cancers.	
7. Suffering from major trauma or surgery within half a year.	
**For health participants**	**For NAFLD patients**
1. Present fatty liver examined by abdominal ultrasonography.	1. A history of excessive daily alcohol consumption (< 20 g for women and < 30 g for men).
2. Previous confirmed liver diseases including viral hepatitis, alcoholic fatty liver, autoimmune liver disease and Wilson' s disease.	2. Suffering from other liver diseases and other abnormal condition to induce hepatic steatosis such as viral hepatitis, autoimmune hepatitis, drug-induced hepatitis, Wilson's disease, Cushing's syndrome and hypothyroidism.

Supplemental figures and legends

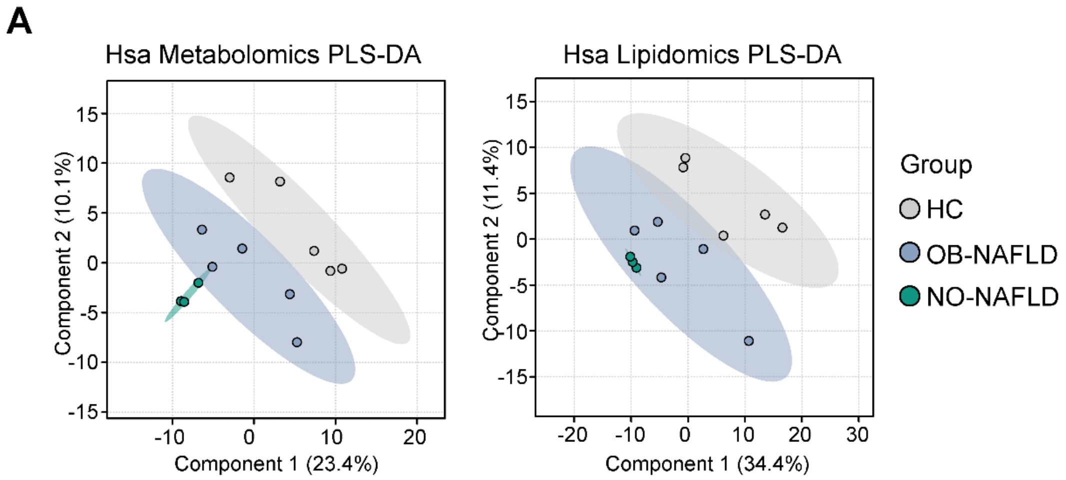

Figure S1 Serum metabolomics and lipidomic analyses confirmed distinctions in the metabolomic profiles between the NO-NAFLD and OB-NAFLD groups. PLS-DA: Partial Least Squares Discriminant Analysis.

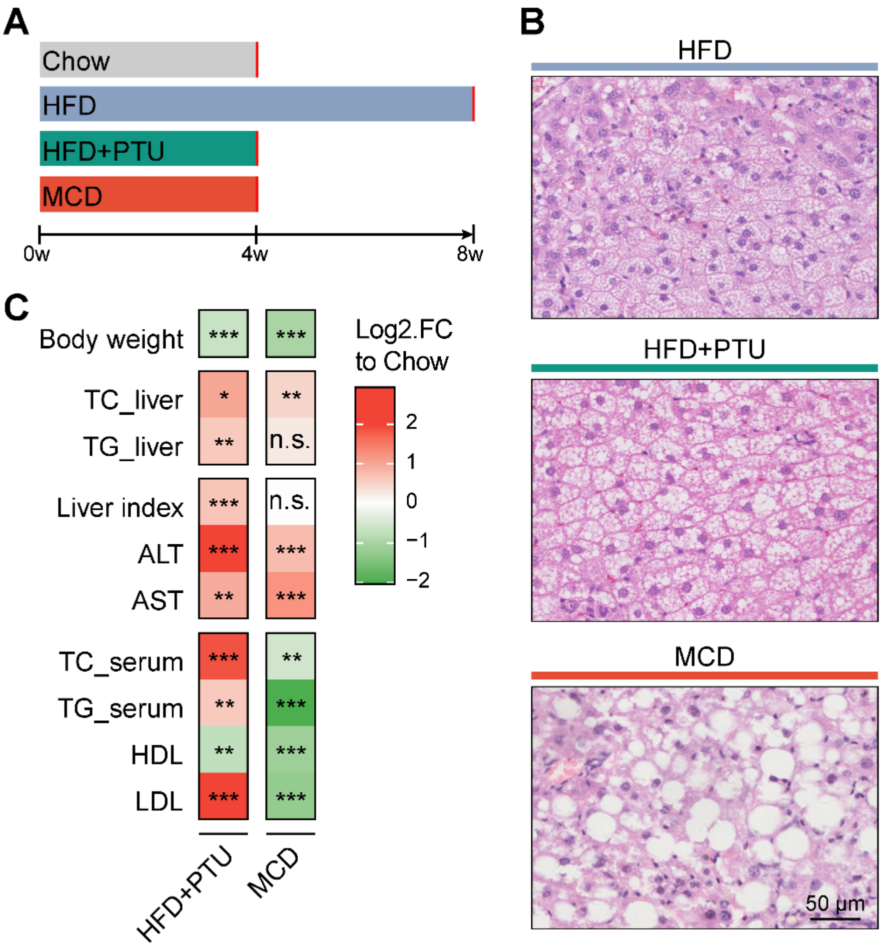

-Figure S2 NAFLD was developed in the MCD-induced mouse model without featuring systemic lipid abnormalities. (A) Schematic diagram of the process for establishing the MCD-induced mouse model; (B) Histological features of hepatic tissue sections stained with Hematoxylin and eosin (H&E); (C) Body weight and biochemical measurements of the models compared to the control, with red indicating upregulation and green indicating downregulation. Statistical significance in the figures is denoted as follows: Compared to the control group: n.s. ($P \geq 0.05$, no significant difference), * ($P < 0.05$), ** ($P < 0.01$), *** ($P < 0.001$).

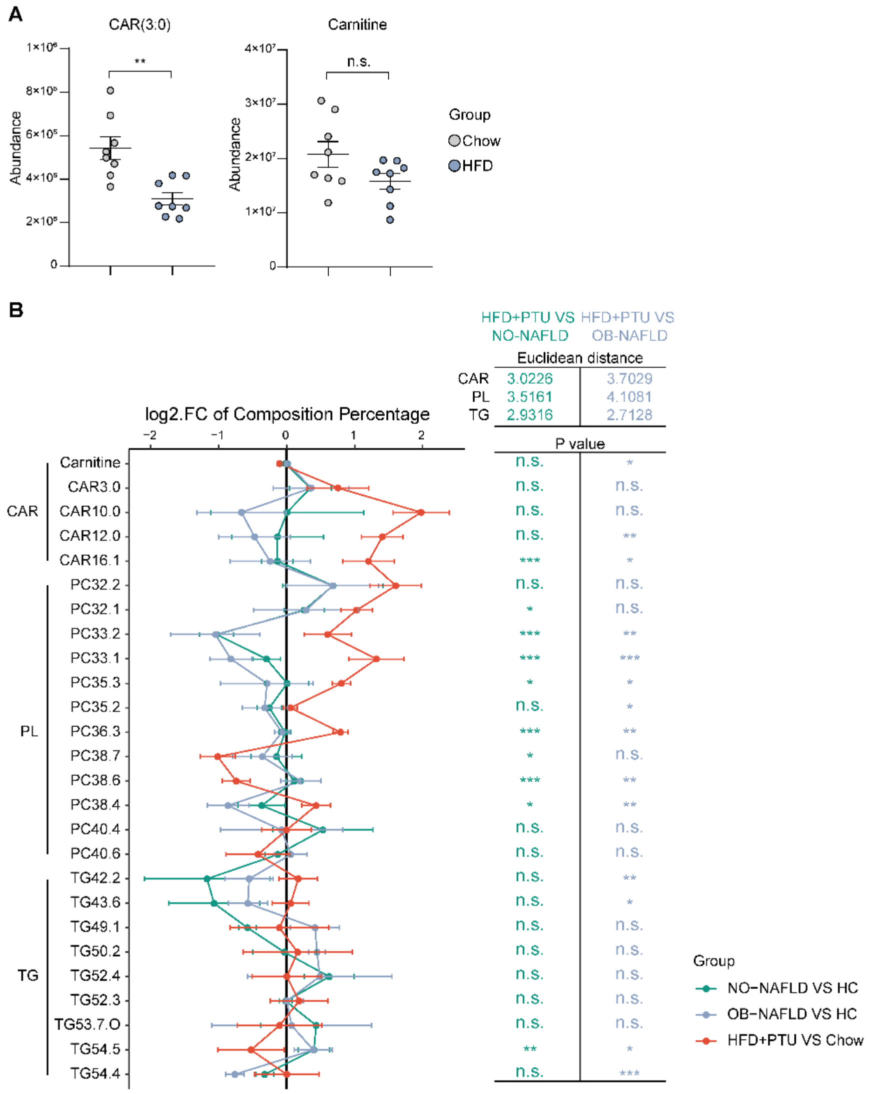

Figure S3 The linkage between the HFD+PTU-induced model and NO-NAFLD subjects as shown by distinctive metabolites. (A) Abundance of serum CAR (3:0) and carnitine in the HFD+PTU model compared to the chow group; (B) Altered composition percentage of distinctive metabolites in the HFD+PTU-induced model and clinical NAFLD subjects compared to their respective controls. The correlation between the model and clinical subjects was assessed using Euclidean distance and a two-tailed Student's t-test. Statistical significance: * ($P < 0.05$), ** ($P < 0.01$), *** ($P < 0.001$), and n.s. (non-statistical significance).

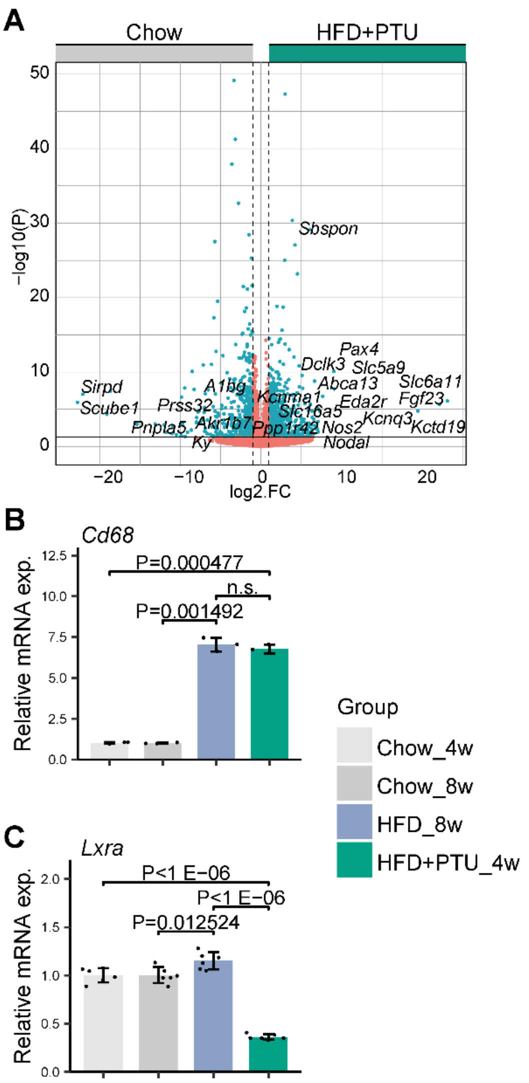

Figure S4 Comparative metabolomics of liver in HFD+PTU induced model and HFD-induced model. (A) RT-qPCR results showing differences in the expression of ***Cd68***; (B) RT-qPCR results showing differences in the expression of ***Lxra***. Statistical significance was calculated using one-way ANOVA followed by Tukey’s test.
